# Comparison between the socio-educational profiles of children with verbal and non-verbal Autism Spectrum Disorder

**DOI:** 10.1590/2317-1782/20232021317en

**Published:** 2023-10-09

**Authors:** Simone Aparecida Lopes-Herrera, Daniela Gisley de Sousa Costa, Thaís Rosa dos Santos, Aline Martins

**Affiliations:** 1 Departamento de Fonoaudiologia, Faculdade de Odontologia de Bauru, Universidade de São Paulo - USP - Bauru (SP), Brasil.; 2 Graduação em Fonoaudiologia, Faculdade de Odontologia de Bauru, Universidade de São Paulo - USP - Bauru (SP), Brasil.; 3 Programa de Pós Graduação em Fonoaudiologia, Faculdade de Odontologia de Bauru, Universidade de São Paulo - USP - Bauru (SP), Brasil.

**Keywords:** Autism, Communication, Cognition, Development, Language

## Abstract

**Purpose:**

Compare the psychoeducational profiles of children with verbal and non-verbal Autism Spectrum Disorder (ASD).

**Methods:**

Cross-sectional study conducted with a sample of 30 children with a medical diagnosis of ASD (15 verbal and 15 non-verbal) aged 2-9 years. The Psychoeducational Profile-Revised (PEP-R) scale was applied to assess the children’s development. The data were analyzed quantitatively and comparatively. Analysis of covariance (ANCOVA) was performed to evaluate the compatibility between the groups regarding the scores obtained in each PEP-R area, with chronological age as the covariate, and Student’s t-Test was used for the independent samples (p≤0.001).

**Results:**

The scores in the different areas of the PEP-R were higher in the verbal group, with associations between language development and cognitive and social adaptive skills in the studied sample. Comparison between the groups showed a lower profile of the non-verbal group, with statistically significant differences in the areas of imitation, perception, gross and fine motor coordination, eye-hand coordination, cognitive performance, and verbal performance.

**Conclusion:**

The goal of comparing the psychoeducational profiles of verbal and non-verbal ASD children was reached, and statistically significant differences were observed. The children with non-verbal ASD presented a lower psychoeducational profile compared with that of verbal ASD children. Further studies with larger samples, delimited age groups, and more specific tests in each developmental area are suggested.

## INTRODUCTION

The Diagnostic and Statistical Manual of Mental Disorders-Fifth Edition (DSM-5) defines Autism Spectrum Disorder (ASD) as a neurodevelopmental disorder characterized by atypical development with different levels of disability severity, resulting in the impairment of social skills and verbal and non-verbal communication skills, in addition to the manifestation of restricted behaviors and interests, with the presence of repetitive and stereotyped movements^(1)^. ASD presents a multifactorial origin, thus requiring evaluation and understanding of all aspects related to the individual’s genetic, social, and emotional characteristics. The outline of the psychoeducational profile of a child with ASD is essential for prognosis, and it should be associated with the planning of intervention. This includes considering their deficits in fine motor skills, sensory coordination, language delay, and intellectual disability^(2)^.

The onset of ASD symptoms tends to occur very early. Families report that most of its characteristics are noticeable in the child’s day-to-day life and can be developed to a greater or lesser extent depending on environmental factors. The social interaction deficit may manifest itself more noticeably when there is an increase in the child’s social circle (such as with the child’s participation in the school environment), and it is evidenced in difficulty in socializing and impaired interaction with other interlocutors. Regarding communication, it is possible to notice manifestations of cases ranging from delayed development of speech and language to speech with limited communicative functions. Concerning behavior, the establishment of a consistent routine can be observed, in addition to stereotypies and repetitive behaviors that are individually manifested in different degrees and forms, but characterized by pattern repetitions and apparent lack of functionality. The consequences of the disorder may be milder or more severe, according to the level of manifestation and functioning of the person with ASD at a given time^(3)^.

An interdisciplinary approach becomes indispensable, from diagnosis to intervention, with a team of physicians, psychologists, educators, psycho-educators, occupational therapists, and speech-language pathologists, as it assists in developing verbal and non-verbal communication, socialization, sensory integration, literacy, and other skills depending on the disorder level, as well as the intensity and adequacy of treatment^(4)^.

Throughout the language acquisition process in children with typical development, some individual variations are observed both in the acquisition itself and in the speed and quality at which the language is acquired; therefore, it is a complex process determined by a range of factors, from neuropsychological maturity, affectivity, and cognitive development to the child’s living environment^(5)^.

Furthermore, the child’s full development is not solely based on biological conditions, but also on environmental or socio-environmental factors, such as stimulation, socioeconomic status, and family setting. A very common correlation often established in the literature is that cognitive development is associated with linguistic acquisition. Consequently, the better the language development, the more adept the children would be at communicating their thoughts, feelings, ideas, and intentions, as well as at understanding the same processes in others. According to this premise, language and cognition would be closely linked^(6)^.

Regarding ASD, the cognitive issue cannot be associated only with the individual’s intelligence quotient, nor be measured through psychometric tests that use orality directly, since most studies that have compared groups of people with non-verbal and minimally-verbal ASD with groups of people with verbal ASD do not include the intelligence quotient as a reliable measure directly related to whether an individual with ASD is considered verbal or non-verbal^(7-11)^.

However, it cannot be denied that language and speech development is correlated to a complex series of cognitive, perceptual, and linguistic skills whose genesis relies on the pre-verbal period. The symbolic construction is part of the cognitive set of skills that are essential for the formation of the linguistic sign and, therefore, for the use of words as a form of expression. Thus, the development of symbolism is directly associated with the progress of oral language^(12)^.

Communication impairment in ASD cases affects both verbal and non-verbal skills at varying degrees given the heterogeneity of this condition. The ASD population often has an absent or inconsistently established repertoire of verbal elements (words and phrases), especially in early developmental stages. Some children never flourish their communication skills, with a total absence of speech; others have poorly developed language, which can be characterized by jargon, echolalia, pronominal inversions, abnormal prosody, and monotonous intonation. Those who acquire verbal skills may demonstrate persistent deficits in establishing a conversation, such as a lack of social reciprocity and functionality in their communication^(13)^.

According to the literature^(10,11,14-20)^, many researchers tend to adopt definitions regarding what would be considered a child with verbal, minimally verbal, and non-verbal ASD^(11)^ and, even though there is no consensus to date regarding the definition of most authors, given the heterogeneity of intellectual functioning and linguistic skills among these individuals with ASD, some parameters should be considered both based on direct observation and application of expressive and receptive language tests and protocols, as well as on video analysis of communicative situations along with parental reports. Subjects with ASD that are considered verbal (V) in most of the studies are those who produce varied functional phrases in more than one communicative context in their linguistic repertoire^(10,14-16)^.

As for the minimally verbal (MV) individuals, there is a strong discussion in the literature concerning whether they should be classified as verbal (V) or non-verbal (NV); many authors^(10,14,16,18)^ have argued that MV individuals are those able to functionally use at least a small repertoire of isolated words (from 10 to 30) in their communication and/or fixed words and phrases in one or more communicative interaction contexts^(15-17)^. Conversely, NV individuals are those who have not developed any functional verbal language or remain minimally verbal, without expansion of their communicative repertoire after 12 years of age (early adolescence), although some authors have suggested that age should be ≤7 years^(11,16,18-20)^. As aforementioned, there is no consensus among researchers in the area, which means that—when conducting a study on this theme—authors should take a clear stance on which definition they will be using, which is not always the case, hindering the reliability of meta-analysis and systematic literature reviews.

From the perspective of the aforementioned authors^(10,11,18-20)^, to have a more comprehensive understanding of the clinical characteristics and neurobiological correlates of this subgroup (V and MV) of individuals with ASD, it would be appropriate to define them using very strict diagnostic criteria and conduct research in the area to specify with increasing detail which criteria are being used to define the experimental and control groups with these populations.

The Psychoeducational Profile-Revised (PEP-R)^(21,22)^ was specifically designed to assess children with ASD or further related communication disorders. This instrument was created because of the need to identify irregular learning patterns, providing valuable information to develop individualized psycho-educational planning according to the principles of the Treatment and Education of Autistic and Related Communication Handicapped Children (TEACCH) model^(21)^. In addition, professionals who work with this model often use the PEP-R in their evaluations and clinical or educational reassessments. Nevertheless, other theoretical lines of behavioral or developmental basis also use this instrument. It has already been translated and validated into Brazilian Portuguese and has been used as a reference^(22)^ in many studies and clinical applications in Health and Education, specifically for assessing the development of children with ASD^(23-26)^.

Brazilian studies validating the PEP-R^(22,23)^ present the psychometric properties of the Brazilian version of this instrument, which evaluates the developmental age in children with ASD or related communication disorders. The criterion validity of the Development and Behavior dimensions of the scale was evaluated by administering the PEP-R to 20 children with ASD, 20 children with Down Syndrome, and 40 children with typical development, aged 4-9 years. The Raven’s Colored Progressive Matrices Tests and the Brazilian version of the Child Behavior Checklist (CBCL) were also administered. The correlations between the PEP-R and these assessments were 0.54 and 0.39, respectively. Reliability among evaluators (W-Kendall) ranged between 0.80 and 0.87, with internal consistency between 0.80 and 0.97. Comparison of the final raw scores of the PEP-R Behavior scale in the three groups confirmed the discriminant validity of the instrument, showing that the group with ASD presented greater impairment in the investigated dimensions compared with those of the other groups.

A later study^(25)^ using the PEP-R found that children with ASD aged 1 year and 3 months to 2 years and 11 months presented a lack of development in the areas of cognitive performance and verbal performance, probably because these areas include skills directly associated with speech repertoire.

In general, studies involving PEP-R^(22-26)^ assess the levels of developmental functioning and abnormalities in the behavior of children, especially those diagnosed with ASD, allowing the construction of an educational plan that acknowledges both the acquired skills and those still under development. Understanding the differences and the functioning stages of skills related to development in V and NV individuals provides the professionals who work with them with an orientation to their intervention practices, redirecting strategies and tracing differentiated planning, with different priorities suited for each profile, focusing on the improvement of each skill, which consequently becomes a requirement for the development of other skills^(25)^. Knowing the limitations of each profile (V and NV) corroborates the implementation of differentiated models of action, further contributing to the clinical assessment and considerations regarding prognosis.

In conclusion, this study aimed to compare the psychoeducational profile of children with verbal and non-verbal ASD. The hypothesis proposed herein is that children with verbal ASD would present a superior performance in their psychoeducational profile than those with non-verbal ASD, considering the chronological order of the mastery and development of certain skills, which occasionally are requirements to further acquisitions, targeting mainly orality (verbal *vs*. non-verbal relationship). At all times, researchers have focused on specifying— among the studied population— in which skills children with verbal ASD would have a superior performance—and in which skills their performance would be equal or analogous to that of children with non-verbal ASD, for a better understanding of the challenges between the multiple development domains in this population.

## METHODS

This study was conducted at the College of Dentistry of Bauru, University of São Paulo (FOB-USP). The fundamental ethical principles that guide research involving human beings, as described and established by Resolution No. 466/12 of the National Health Council (CNS) and its complements were considered in all stages of this study, which was approved by the Ethics Research Committee of FOB-USP under protocol No. 4.588.003.

An interdisciplinary team evaluated 30 individuals with a clinical medical diagnosis of ASD without associated conditions, respecting the diagnostic criteria of the Diagnostic and Statistical Manual of Mental Disorders (DSM-5)^(1)^ and the International Statistical Classification of Diseases and Related Health Problems (ICD-10)^(27)^ effective at the time of data collection, amounting to 15 verbal (Group 1) and 15 non-verbal (Group 2) children aged 2 years and 3 months to 9 years and 3 months.

To define which children would be considered verbal and which would be considered non-verbal, regardless of the lack of consensus in the literature - as previously mentioned in the Introduction section, this study considered the criteria proposed and used by authors related to the theoretical framework used in similar studies^(10,11,14-20)^ and a recent literature review on the clinical and neurobiological characteristics of children with minimally verbal ASD^(11)^. This methodology showed that most studies comparing groups of people with non-verbal and minimally verbal ASD with groups of people with verbal ASD have not acknowledged the intelligence quotient as a reliable measure directly related to whether an individual with ASD is considered verbal or non-verbal^(7-11)^, which also explains why this factor was not considered in the sample of this study.

Although most authors have not researched a consensus on defining verbal, minimally verbal, and non-verbal children with ASD, it is important to emphasize that, given the heterogeneity in intellectual functioning and linguistic skills among these individuals, the following definitions (based on the parents’ reports and videos recorded during the data collection stage) were adopted for the study groups:

***Group 1*** - considered the “verbal” group, included:

- Children with verbal ASD (V): children who produced varied functional phrases in more than one communicative context in their linguistic communication repertoire at the time of data collection^(10,14-16)^;
- Children with minimally verbal ASD (MV): children who functionally used at least one small repertoire of isolated words (from 10 to 30) in their communication and/or fixed words and phrases in one or more communicative interaction contexts^(10,14-18)^ at the time of data collection.

***Group 2*** - considered the “verbal” group, included:

- Children with non-verbal ASD (NV): children who did not develop any functional verbal language or remained minimally verbal, with no expansion of their communicative repertoire^(11,16,18-20)^ at the time of data collection.

All participants along with their parents and/or legal guardians completed and signed an Informed Consent Form (ICF) before study commencement.

The Psychoeducational Profile-Revised (PEP-R) (version translated to and validated for Brazilian Portuguese)^(21)^ was applied to collect the data^(22)^, enabling the analysis of aspects related to the developmental and socio-educational profiles of the children with ASD. The PEP-R was administered by a speech-language pathologist qualified to apply this test and every application was recorded.

The PEP-R^(21,22)^ is an instrument applicable to preschool children aged 2-12 years that measures the developmental age, identifying irregular and idiosyncratic learning patterns of children with ASD. It is a widely used instrument in both international and national studies in the field of ASD. It has been validated for Brazilian Portuguese^(22,23)^ and presents psychometric properties that enable confirmation of its discriminant validity.

The PEP-R instrument includes two evaluation scales: one related to Development and the other to Behavior. There are seven development areas used in the study, namely: gross motor skills (18 items), fine motor skills (16 items), visual motor integration/eye-hand coordination (15 items), perception (13 items), imitation (16 items), general cognitive performance (26 items), and verbal performance/language (27 items), totaling 131 evaluated items. The areas of imitation, language, and cognition are directly associated with the overall development of the individual. For each area, a specific scale was designed with tasks to be performed or behaviors to be observed, with a varying number of items—as above mentioned—per development area.

The PEP-R application kit consists of a series of toys and educational materials to be presented to the child in a playful and structured way as a game and play. The test was carried out in the institution at pre-selected times according to the availability of the families and children, being conducted individually in a large, well-lit, silent evaluation room available on site. For each child, the test was completed in a single session and properly recorded on a video no longer than 90 minutes, with an interval if necessary (minimum time of 60 minutes). The researcher, the speech-language pathologist that administered the test, and the child were present during the applications.

For a better test application, the items are arranged according to development, that is, from the simplest activities to the most complex ones. Standardized verbal instructions should be avoided when providing the command to perform the activities. The PEP-R evaluator’s manual provides specific application techniques, such as the predominance of verbal instructions, the use of simplified language to guide the child, the use of tips and gestures to signal what is requested from the child, demonstrations of how the activity should be performed, and physical guidance during tasks, such as helping with the materials or moving the child’s hands toward them. At the end of the session, the scores obtained are distributed in the areas of development and behavior (in the case of this study, only the development area was used).

Scores are classified in three different ways in the developmental scale: Passed (P), when the child performs the task correctly, that is, successfully (for which 1 point is assigned); Emergent (E), when the child demonstrates some type of knowledge to perform the task, but cannot fully perform or complete it (for which 0.5 point is assigned); and Failed (F), when the child does not demonstrate knowledge to perform the activity even after numerous demonstrations, consequently not performing the task (no points assigned).

The data was then computed and quantitatively analyzed by ANCOVA, which evaluated the compatibility between the groups regarding the scores obtained in each area using chronological age as a covariate. This analysis allows an “adjustment” to a response variable effect that might have been influenced by other variables or uncontrolled variations (such as the broad age range). In addition, the Student’s *t*-test was applied to the independent samples. A significance level of 5% (*p*≤0.001) was adopted for all statistical analyses.

Application of ANCOVA is recommended for quasi-experiment studies, that is, when there are difficulties in obtaining similar experimental measurements, as in the present case. ANCOVA may include one or more quantitative variables that are related to the sought results, and these variables are included in the analysis because of the influence they have on the outcome - they are known as covariates. Through the use of covariates in the ANCOVA model, it is possible to eliminate the systematic errors that occur out of the researcher’s control and that can often bias the results and explain the differences in responses due to the participants’ characteristics.

## RESULTS

The PEP-R has psychometric parameters to trace the children’s developmental age according to the score obtained in each area of the development scale. It is important to emphasize that analysis of the complete sample showed a difference in chronological age between the verbal and non-verbal groups, which rendered pairing impossible. The Student's *t*-test was applied to the independent samples aiming to compare the two groups in terms of chronological age to confirm the significant difference between the ages of the groups, corroborating the hypothesis that there is a statistically significant difference between the ages (*t*=-2.503 and *p*=0.018). Therefore, a limitation was verified in the analysis of the study results and to correct this event for the comparison between groups, the ANCOVA was performed with chronological age as the covariate, as explained in the Method section.

Table 1 shows the chronological and developmental ages of each participant. It is noteworthy that the chronological age and the developmental age attributed by the PEP-R are numerically represented in months. Thus, it is possible to observe that all the children with ASD evaluated, both verbal and non-verbal, presented a developmental age below their chronological age; the differences between these ages ranged from 19 to 108 months (approximately 1 year and 6 months to 9 years) in the verbal group from 18 to 92 months (approximately 1 year and 5 months to 7 years and 6 months) in the non-verbal group.

**Table 1:** Difference between Chronological Age and Developmental Age defined by the PEP-R in Groups 1 and 2

Verbal Group (G1)				Non-Verbal Group (G2)			
Participant	Chronological	Developmental	Difference	Participant	Chronological	Developmental	Difference
	Age	Age			Age	Age	
1	66	42	24	1	41	9	32
2	81	45	36	2	27	7	20
3	85	60	25	3	54	22	32
4	57	20	37	4	46	12	34
5	41	22	19	5	37	12	25
6	66	22	44	6	29	9	20
7	132	24	108	7	109	17	92
8	64	41	23	8	60	18	42
9	48	20	28	9	49	16	33
10	50	22	28	10	45	22	23
11	46	22	24	11	33	4	29
12	53	32	21	12	68	16	52
13	65	28	37	13	69	18	51
14	83	55	28	14	24	6	18
15	111	36	75	15	33	14	19
Average	69.87	25.20	44.13	Average	48.27	21.27	27.00
Standard Deviation	25.73	8.86	26.24	Standard Deviation	21.96	8.48	23.66

**Statistical tests adopted** - analysis of covariance (ANCOVA) and Student’s *t*-test for independent samples, significance level (*p*≤0.001).

**Caption:** Chronological Age = shown in months; Developmental Age = defined in months by the PEP-R; Difference = difference between the chronological age and the age defined in months by the PEP-R.

Table 2 shows that the *p-values of the ANCOVA were significant in all areas of the PEP-R, confirming the hypothesis that the performance of individuals with verbal ASD was higher than that of those with non-verbal ASD in all of these areas. In addition, as a general observation, it is evident how the overall averages in all areas of development are higher in the verbal group compared with those of the non-verbal group, with a significant *p-value in the ANCOVA (Tables 2-5).

**Table 2:** Average and analysis of covariance (ANCOVA) expressed by *p*-value based on the alternative hypothesis (H) that the performance of the Verbal Group (G1) is superior to that of the Non-Verbal Group (G2)

Evaluated item	Average				*p*-value
	**Verbal Group (G1)**		**Non-Verbal Group (G2)**		H=G1>G2
	**ObtV**	**MaxV**	**ObtV**	**MaxV**	
Imitation	10.27	16	1.67	16	**0.000011**
Perception	10.73	13	6.07	13	**0.000088**
FineM	9.13	16	4.20	16	**0.009853**
GrossM	13.40	18	6.60	18	**0.000083**
Eye-Hand	8.60	15	3.73	15	**0.008168**
GeneralCo	12.67	26	2.33	26	**0.000403**
VerbalCo	9.87	27	1.07	27	**0.000408**
TotalS	74.67	131	25.67	131	**0.000049**

**Statistical test adopted -** ANCOVA model, significance level (*p*≤0.001).

Captions: ObtV = value obtained in the item by the child at the time of data collection; MaxV = maximum possible value in the evaluated item according to the protocol; FineM = Fine Motor Skills; GrossM = Gross Motor Skills; GeneralCo = General Cognitive Performance; VerbalCo = Verbal Cognitive Performance/Language; TotalS = Total Score; H=G1>G2 = Alternative hypothesis (verbal group has a higher performance than the non-verbal group).

**Table 5:** Comparison of the performances of the Verbal Group (G1) X Non-Verbal Group (G2) in the areas of General Cognitive Performance (General C.) and Verbal Cognitive Performance (Verbal C.) of the PEP-R

Participant	Verbal Group		Non-Verbal Group	
	General C.	Verbal C.	General C.	Verbal C.
1	17	19	2	1
2	17	13	0	1
3	26	23	7	2
4	4	5	1	1
5	4	4	1	0
6	6	3	0	0
7	12	3	4	1
8	18	10	2	1
9	4	5	2	4
10	12	5	7	2
11	4	2	0	0
12	14	10	2	1
13	10	13	6	1
14	23	18	1	0
15	19	15	0	1
Average	12.67	9.87	2.33	1.07
Standard Deviation	7.29	6.7	2.49	1.03
Interval (min-max)	4-26	2-23	0-7	0-4
			(*p*) General C	**0.000403**
			(*p*) Verbal C.	**0.000408**

**Statistical tests adopted -** ANCOVA model and Student’s *t*-test for independent samples, significance level (*p*≤0.001).

Captions: Interval (minv - maxv) = minimum and maximum scores obtained in each evaluated area.

Analyzing the variable Imitation separately (Table 3), it is important to highlight that this area contains activities that assess the child’s ability to perform verbal or motor imitations; usually, the imitation score in verbal individuals ranged from 2 to 16 points, while the score of children with non-verbal ASD ranged from 0 to 8 points. On the other hand, the variable Perception, also shown in Table 3, refers to the area that evaluates the functioning of sensory, visual, and auditory modalities. The results indicate that, in general, the score of the verbal group was higher (9-13 points) than that of the non-verbal group (2-11 points).

**Table 3:** Comparison between the performances of the Verbal Group and Non-verbal Group in the areas of imitation and perception of the PEP-R

Participant	Verbal Group		Non-Verbal Group	
	Imitation	Perception	Imitation	Perception
	15	12	1	3
2	15	10	0	3
3	16	13	8	10
4	6	9	2	5
5	7	9	2	8
6	9	9	1	3
7	13	10	1	8
8	13	11	1	9
9	2	10	1	6
10	5	11	7	7
11	5	10	0	2
12	11	12	0	7
13	9	11	1	11
14	15	13	0	3
15	13	11	0	6
Average	10.27	10.73	1.67	6.07
Standard Deviation	4.46	1.33	2.47	2.84
Interval (minv - maxv)	2-16	9-13	0-8	2-11
			(*p*) imitation	**0.000011**
			(*p*) perception	**0.000088**

**Statistical tests adopted -** ANCOVA model and Student’s *t*-test for independent samples, significance level (*p*≤0.001).

Captions: Interval (minv - maxv) = Minimum and maximum scores obtained in each evaluated area.

The Gross Motor Skills and Fine Motor Skills areas of the PEP-R include activities such as walking alone, climbing stairs, and keeping balance on one foot (gross motor assessment) as well as using scissors and twisting off a bottle cap (fine motor assessment). Table 4 shows the results obtained in the motor areas. The scores of the verbal group were higher than those of the non-verbal group. Regarding gross motor skills, the results of individuals with verbal ASD ranged from 9 to 18 points, whereas those of children with non-verbal ASD varied between 2 and 12 points. As for the fine motor skills, the results of the children with verbal ASD ranged from 5 to 16 points and those of the children with non-verbal ASD ranged from 0 to 10 points.

**Table 4:** Comparison between the performances of the Verbal Group and Non-verbal Group in the areas of Fine Motor Skills, Gross Motor Skills, and Visual Motor Integration/Eye-hand coordination of the PEP-R

Participant	Verbal Group			Non-Verbal Group		
	Fine Motor	Gross Motor	Visual Motor	Fine Motor	Gross Motor	Visual Motor
	9	14	10	4	3	1
2	13	17	13	1	2	1
3	16	18	15	10	11	8
4	6	12	6	7	9	3
5	5	12	5	1	4	2
6	11	9	4	1	6	0
7	6	13	4	2	10	4
8	14	17	10	10	7	6
9	8	10	6	3	7	5
10	6	10	5	10	12	8
11	5	13	6	0	2	0
12	10	15	8	4	7	6
13	6	13	10	7	10	7
14	14	18	15	0	2	1
15	8	10	12	3	7	4
Average	9.13	13.4	8.6	4.2	6.6	3.73
Standard Deviation	3.68	3.04	3.85	3.69	3.4	2.84
Interval (min-max)	5-16	9-18	4-15	0-10	2-12	0-8
					(*p*) Fine Motor	**0.009853**
					(*p*) Gross Motor	**0.000083**
					(*p*) Visual Motor	**0.008168**

**Statistical tests adopted** - ANCOVA model and Student’s t-test for independent samples, significance level (*p*≤0.001).

Captions: Interval (minv - maxv) =Minimum and maximum scores obtained in each evaluated area.

Table 4 also presents the scores referring to the Visual Motor Integration/Eye-hand Coordination area, which is composed of activities such as writing on paper, coloring inside the lines, drawing and copying figures, as well as stacking several blocks. The scores obtained by the group of children with verbal ASD (4-15 points) were higher than those achieved by the children with non-verbal ASD (0-8 points).

The General Cognitive Performance and Verbal Cognitive Performance/Language areas in the PEP-R instrument have maximum scores of 26 and 27 points, respectively. These areas are centered on general and verbal cognition/language. General cognitive performance activities require some receptive understanding of language to be successfully executed. The main difference between these two areas is that general cognitive performance items comprise practical activities that do not require expressive language, while verbal cognition items prompt a verbal response. Table 5 shows the significant difference between the performance of individuals with verbal and non-verbal ASD in both areas. Concerning general cognitive performance, the results of individuals with verbal AD ranged from 4 to 26 points and those of individuals with non-verbal ASD varied between 0 and 7 points. As for verbal cognition performance, the results ranged from 2 to 19 and from 0 to 4 points for the children with verbal and non-verbal ASD, respectively.

## DISCUSSION

Overall, the psychoeducational profiles of children with verbal and non-verbal ASD were compared, confirming the main hypothesis of this study that children with verbal ASD presented—in the analyzed sample—a performance superior to that of children with non-verbal ASD.

The developmental view of areas of mastery assessed by items associated with specific behaviors considers the chronological order of mastery and development of certain skills that are occasionally required to acquire other skills, focusing mainly on orality (verbal vs. non-verbal).

The main contribution of the results of this study was to analyze and specify in which skills children with verbal ASD—in the study sample—would have a superior performance, and in which skills their performance would be equal or analogous to that of children with non-verbal ASD, for a better understanding of the challenges between the multiple development domains in this population, with future impacts on the design of clinical and educational interventions.

Nonetheless, there are important limitations to be discussed. First, it is necessary to consider that some of the individuals in the verbal group presented a chronological age higher than those in the non-verbal group, which may have contributed to the aforementioned difference in many of the evaluated item scores, although statistical tests were applied in the comparative analysis to mitigate this difference.

As the age range of the sample in this study is 2 years and 3 months to 9 years and 3 months (that is, between 27 and 111 months), the older the children, the better their performance: more effective use of communicative skills, which corroborate the findings of studies on language^(6-8)^. The age range of the assessed children—3 years and 5 months to 9 years and 3 months (41 to 101 months) in the verbal group and 2 years and 3 months to 9 years and 1 month (27 to 109 months) in the non-verbal group—may have influenced the results of this study even with the application of ANCOVA. This is an important limitation of this study since the age scope of its sample precluded the equal pairing of the results by age, allowing it to be done only by group.

The definitions of children with verbal, minimally verbal, and non-verbal ASD are relevant factors to be considered in the analysis and discussion of the results as they present a limitation in use, mainly because this is not an objective criterion, and although this study was based on the definitions used by several renowned authors in the area, they also discuss this topic^(10-20)^. According to these authors, to have a more comprehensive understanding of the clinical characteristics and neurobiological correlates of the verbal group (individuals with verbal and minimally verbal ASD), it would be appropriate to define them using very strict diagnostic criteria. They also state that studies in this area should specify with increasing detail the criteria being used to define their experimental and control groups. Therefore, the definition of each of the study groups was presented, so that the analysis performed could comply with them.

The decision to describe and discuss each evaluated area was made based on the insufficiency of descriptive studies in this format and to provide information so that professionals who work with ASD can use these data as a reference—when choosing to follow approaches similar to the one presented here—to analyze the possible relations between the areas they want to focus on in future evaluations and interventions with their target populations. For example, speech-language pathologists may be more attentive to some of their patients regarding the relations between imitation and verbal performance (especially in preschool children or at a pre-verbal level) and, at other times or for other patients (e.g., pre-literacy ages), they may focus on perception, fine motor skills, and visual motor integration/eye-hand coordination. In other words, the main contribution of the results presented here is to allow a differentiated approach—using the same instrument—area by area in two or more skills or in the general psychoeducational profile provided for the two evaluated groups (verbal and non-verbal).

The Imitation area of the PEP-R, which is responsible for assessing the child’s ability to provide a verbal or motor imitation of something, is of paramount importance for the development of individuals with ASD because of the fundamental relation between imitation, language, and learning, given that, to be able to learn words, children must have the basic skills necessary to imitate, such as joint and shared attention, among other^(2,5)^. Imitation also plays a key role in socialization, because through it children learn the strategies of behavior, cooperation, coordination, and response to interactions with other people.

Items in the PEP-R Imitation area include imitation of broad body movements, use of certain materials, and sounds and words, all demonstrated by the evaluator with a subsequent direct imitation request. Because individuals with non-verbal ASD have difficulty demonstrating or understanding verbal behaviors (limitation of receptive/expressive language), they may present losses in their ability to imitate, mainly sounds and words. This fact may have impacted the performance of the non-verbal group in this area. Similarly, children in the verbal group who were able to make more effective use of receptive and expressive communicative skills tended to perform the imitation function more easily, which would justify the higher score obtained by this group compared with that of the non-verbal group.

A study^(15)^ addressing the communicative skills of individuals with non-verbal ASD also reported performance deficits, such as decreased intensity and frequency of some adaptive behaviors, differences in oral receptive vocabulary, pragmatics with significant changes in the total number of communicative acts and utterance per minute, reduction of communicative acts in imitation, and communication with a predominance of gestures. The data on the pragmatic profile have corroborated those of other studies^(6,8,14,16)^.

As for the Perception area, which examines the ability to select and organize a given stimulus, children with ASD often present difficulty in storing and integrating sensory information; therefore, it is important to pay attention to the performance limitations in this area. As a result of these perceptual difficulties found in individuals with ASD, most of the items in the perception area of the PEP-R were formulated for younger children ^(21, 22)^, with activities such as visual following of soap bubbles, observation of figures in a book, and orientation by sounds. Even so, the results obtained in the perception area in this study show that the overall score of the verbal group was higher than that of the non-verbal group, with the latter presenting greater difficulties in performing these items.

As for the motor skill areas, it is known that gross or global motor skills are related to the overall control of the body, being responsible for maintaining static and dynamic balance and body posture. In childhood, this motor skill should be the first to be perfected since it is involved in the largest movements of the body, requiring the participation of many body muscles. Furthermore, individuals with severe gross motor impairments tend to present decreased social skills^(16,28,29)^.

Based on the assumption that impairment in gross motor skills leads to decreased social skills, interference with oral communication may be observed, given that social skills include communication. This fact could justify the higher scores in the area of gross motor skills obtained by the verbal group compared with the non-verbal group. A statistically significant difference was observed in the quantitative comparison between the gross motor skills of the verbal and non-verbal groups, with a better performance of the first compared with the latter in this development area.

A previous study^(30)^ reported that motor impairment can interfere with further expressive language skills. This could be one of the justifications for the lower score obtained by the non-verbal group in this area, that is, it suggests the following interrelationship: less developed motor skill, greater impairment in language acquisition and greater difficulties in oral language development. Few studies have associated the development of gross motor skills with the exclusive development of oral expressive language. Therefore, further research is recommended for a more thorough comprehension of this issue.

On the other hand, fine motor skills^(28-30)^ are specifically related to the execution of more precise and refined movements, requiring and resulting from greater control of specific muscles. It is important to note that the greater the degree of complexity required for the execution of an activity, the greater the impact on the cognitive system, and the greater the demand for a precise performance of the central nervous system.

The fine motor skill activities listed in the PEP-R^(21,22)^ include twisting off a bottle cap, using scissors, and threading beads onto a string. These activities are usually mastered in the first 3-4 years of life. Hence, children in this age range or below are still acquiring these skills. The results show that some of the individuals in the non-verbal group are aged up to 4 years, which may have contributed to the lower score on this item obtained by this group compared with those of the verbal group (composed mostly of older children).

A study conducted with children aged 14-15 months^(28)^ pointed out that fine motor coordination may be directly related to language development, because fine motor skills facilitate the interaction between the physical and social domains, thus enabling the development of expressive language. This could explain the superior performance of children with verbal ASD (who present expressive oral language) compared with that of those with non-verbal ASD in this area. However, the fact that some individuals in the non-verbal group are still not in the period of mastery of fine motor skills blurs the conclusion that the performance of the verbal group is superior to that of the non-verbal group in this area. Thus, it cannot be stated that being verbal or non-verbal influenced the fine motor skills of the analyzed sample despite the statistically significant difference found as a result of the limitations of the present study related to the conflicting chronological age between the groups, based on the PEP-R^(21,22)^ and its assessments in this area.

As for the Eye-hand Coordination area, which concerns visual motor integration skills, a study^(29)^ revealed that children with ASD present, from birth, deviant motor characteristics regarding the typical development standards. Motor deficits begin to manifest early in life, before the age of 3. Because eye-hand coordination has the same dependence and interrelationship with the fine and gross motor skills, justification for the better performance of children with verbal ASD compared with that of children with non-verbal ASD in this development area is based on the same reports related to the fine and gross motor skill areas ^(28-30)^. Thus, the results of this study show that the performance of the verbal group was better than that of the non-verbal group in this item.

Regarding the areas of General Cognitive Performance and Verbal Performance—both in the cognitive area of the PEP-R^(21,22)^, which does not require oral expressive language, and in the verbal area, which requires a verbal response—the scores obtained by the verbal group were higher than that achieved by the non-verbal group. Studies^(6,8,14,16)^ addressing the interactive use of communication in children with verbal and non-verbal ASD have revealed that, as far as the use of communicative means by children with ASD is concerned, children with both non-verbal and verbal ASD make great use of gestures to communicate. In this study, children with verbal ASD made greater use of verbal means and poorer use of vocalization than those with non-verbal ASD, similar to what is observed in children with typical development, corroborating the findings of the aforementioned study.

The cognitive performance area of the PEP-R^(21,22)^ has activities that require mainly receptive comprehension of language to be successfully executed. Studies on language associated language acquisition with the development of other cognitive and socio-adaptive skills. The application of ANCOVA to the results of this study confirmed the hypothesis of the superior performance of the verbal group about the non-verbal group. It is known that individuals with ASD present great variability in cognition^(6,7)^. As the PEP-R cognitive development tests^(21,22)^ focus mainly on the receptive and comprehensive skills, the results presented here indicate greater impairment of the comprehension and reception capacities, and not only of (verbal) expression in the non-verbal group. Even so, further research is needed to investigate this information more comprehensively.

Concerning the Verbal Performance area, an initial analysis of the results shows a great difference between the scores of the verbal and non-verbal groups. This was already expected and can be explained by the fact that most PEP-R verbal cognition tests^(21,22)^ require a verbal response. As individuals with verbal ASD have a greater mastery of oral language^(16)^, the fact that the score obtained by the verbal group was significantly higher than that of the non-verbal group is justified, once the individuals with non-verbal ASD who composed the sample presented difficulties regarding oral language acquisition at the time of data collection.

## CONCLUSION

The PEP-R^(21,22)^ indicates the main areas considered essential to the development of children with ASD according to their psychoeducational profile, such as Imitation, Perception, Fine Motor Skills, Gross Motor Skills, Hand-Eye Coordination, General Cognitive Performance, and Verbal Cognitive Performance. The findings of this study show that the socio-educational profile of the individuals with non-verbal ASD analyzed is reduced compared with that of the individuals with verbal ASD in all areas of development evaluated by this instrument.

Thus, the objective of this study to compare the socio-educational profile of children with verbal and non-verbal ASD was achieved, highlighting important differences related to the areas of imitation, perception, gross and fine motor skills, visual motor integration/eye-hand coordination, and global and verbal cognitive performance among children diagnosed with verbal and non-verbal ASD. However, it is important to emphasize that this study has an important limitation: it was not possible to pair the participants by age, which may have directly interfered with the results. Therefore, it is suggested that new studies be conducted to further evaluate these areas to filter and detail the influence of these skills on individuals with verbal and non-verbal ASD, using clear definitions of group differentiation criteria, so that the comparative performance relationship between children with verbal and non-verbal ASD can be based on scientifically proven results and not only on the use of common sense regarding the oral superiority of one group over the other.

Although the findings of this study are relevant, since few studies have compared the performance and the cognitive and intellectual differences between individuals with verbal and non-verbal ASD, it is necessary to continue to research these individuals through the application of other tests as well as with the selection of larger sample sizes, seeking to investigate other aspects of their development and behavior. The importance of age matching in research involving children with verbal and non-verbal ASD is hereby reinforced.

## References

[1] American Psychiatric Association (2013). Diagnostic and statistical manual of mental disorders..

[2] Olivati AG, Assumpção FB, Misquiatti AR (2017). Análise acústica do padrão entonacional da fala de indivíduos com Transtorno do Espectro Autista. CoDAS.

[3] Santos RK, Vieira AMECS (2017). Transtorno do Espectro do Autismo (TEA): do reconhecimento à inclusão no âmbito educacional. Rev. Includere.

[4] Rossi LP, Lovisi GM, Abelha L, Gomide M (2018). Caminhos virtuais e autismo: acesso aos serviços de saúde na perspectiva da análise de redes sociais. Ciênc. Saúde Coletiva..

[5] Carvalho AJA, Lemos SMA, Goulart LMHF (2016). Desenvolvimento da linguagem e sua relação com comportamento social, ambientes familiar e escolar: revisão sistemática. CoDAS.

[6] Zauche LH, Thul TA, Mahoney AED, Stapel-Wax JL (2016). Influence of language nutrition on children’s language and cognitive development: an integrated review. Early Child Res Q.

[7] Munson J, Dawson G, Sterling L, Beauchaine T, Zhou A, Koehler E (2008). Evidence for latent classes of IQ in young children with autism spectrum disorder. Am J Ment Retard.

[8] Rapin I, Dunn MA, Allen DA, Stevens MC, Fein D (2009). Subtypes of language disorders in school-age children with autism. Dev Neuropsychol.

[9] Tager-Flusberg H, Kasari C (2013). Minimally verbal school-aged children with autism spectrum disorder: the neglected end of the spectrum. Autism Res.

[10] Maenner MJ, Shaw KA, Baio J, Washington A, Patrick M, DiRienzo M (2020). Prevalence of autism spectrum disorder among children aged 8 years: autism and developmental disabilities monitoring network, 11 sites, United States, 2016. MMWR Surveill Summ.

[11] Posar A, Visconti P (2021). Update about “minimally verbal” children with autism spectrum disorder. Rev Paul Pediatr.

[12] Damasceno BCE, Leandro VSB, Fantacini RAF (2017). The importance of playing for child development with Down Syndrome. Research Society and Development.

[13] Guerra BT, Almeida-Verdu ACM (2020). Ensino de comportamento verbal elementar por exemplares múltiplos em crianças com autismo. Psicologia (Cons Fed Psicol).

[14] Pickett E, Pullara O, O’Grady J, Gordon B (2009). Speech acquisition in older nonverbal individuals with autism: a review of features, methods, and prognosis. Cogn Behav Neurol.

[15] Kasari C, Brady N, Lord C, Tager-Flusberg H (2013). Assessing the minimally verbal school-aged child with autism spectrum disorder. Autism Res.

[16] Franchini M, Duku E, Armstrong V, Brian J, Bryson SE, Garon N (2018). Variability in verbal and nonverbal communication in infants at risk for autism spectrum disorder: predictors and outcomes. J Autism Dev Disord.

[17] Koegel LK, Bryan KM, Su PL, Vaidya M, Camarata S (2020). Definitions of nonverbal and minimally verbal in research for autism: a systematic review of the literature. J Autism Dev Disord.

[18] Bal VH, Katz T, Bishop SL, Krasileva K (2016). Understanding definitions of minimally verbal across instruments: evidence for subgroups within minimally verbal children and adolescents with autism spectrum disorder. J Child Psychol Psychiatry.

[19] Brignell A, Chenausky KV, Song H, Zhu J, Suo C, Morgan AT (2018). Communication interventions for autism spectrum disorder in minimally verbal children. Cochrane Database Syst Rev.

[20] Koegel LK, Bryan KM, Su PL, Vaidya M, Camarata S (2020). Parent education in studies with nonverbal and minimally verbal participants with autism spectrum disorder: a systematic review. Am J Speech Lang Pathol.

[21] Schopler E, Reichler RJ, Bashford A, Lansing MD, Marcus LM (1990). Psychoeducational Profile Revised (PEP-R)..

[22] Leon V (2002). Estudo das propriedades psicométricas do Perfil Psicoeducacional PEP-R: elaboração da versão brasileira.

[23] León V, Bosa C, Hugo C, Hutz CS (2004). Propriedades Psicométricas do Perfil Psicoeducacional Revisado: PEP-R. Aval Psicol.

[24] Lima F (2016). Análise do instrumento perfil psicoeducacional revisado (PEP-R) para avaliação de crianças com autismo.

[25] Gomes CGS, Souza DG, Silveira AD, Oliveira IM (2017). Intervenção comportamental precoce e intensiva com crianças com autismo por meio da capacitação de cuidadores. Rev Bras Educ Espec.

[26] Bosa CA, Salles JF (2018). Sistema PROTEA-R de avaliação da suspeita de Transtorno do Espectro Autista..

[27] Organização Mundial da Saúde (1996). Classificação Estatística Internacional de Doenças e Problemas Relacionados à Saúde: CID-10.

[28] Hellendoorn A, Wijnroks L, Van Daalen E, Dietz C, Buitelaar JK, Leseman P (2015). Motor functioning, exploration, visuospatial cognition and language development in preschool children with autism. Rev Dev Disabil.

[29] Pusponegoro HD, Efar P, Soedjatmiko, Soebadi A, Firmansyah A, Chen H (2016). Gross motor profile and its association with socialization skills in children with autism spectrum disorders. Pediatr Neonatol.

[30] LeBarton ES, Landa RJ (2019). Infant motor skill predicts later expressive language and autism spectrum disorder diagnosis. Infant Behav Dev..

