# Supplementary material for: Comparison between the socio-educational profiles of children with verbal and non-verbal Autism Spectrum Disorder
Source: Codas. 2023 Oct 9;35(5):e20210317. doi: 10.1590/2317-1782/20232021317en (PMC10688295; doi:10.1590/2317-1782/20232021317en)
Supplement: Translation

# Translation: Comparação do perfil socioeducacional de crianças com Transtorno do Espectro Autista verbais e não verbais

**DOI:** 10.1590/2317-1782/20232021317pt

**Authors:** Simone Aparecida Lopes-Herrera, Daniela Gisley de Sousa Costa, Thaís Rosa dos Santos, Aline Martins

**Affiliations:** 1 Departamento de Fonoaudiologia, Faculdade de Odontologia de Bauru, Universidade de São Paulo - USP - Bauru (SP), Brasil.; 2 Graduação em Fonoaudiologia, Faculdade de Odontologia de Bauru, Universidade de São Paulo - USP - Bauru (SP), Brasil.; 3 Programa de Pós Graduação em Fonoaudiologia, Faculdade de Odontologia de Bauru, Universidade de São Paulo - USP - Bauru (SP), Brasil.

**Keywords:** Autismo, Comunicação, Cognição, Desenvolvimento, Linguagem

## Abstract

**Objetivo:**

Comparar o perfil psicoeducacional de crianças com TEA verbais e não verbais.

**Método:**

Estudo transversal, com amostra de 30 crianças (15 verbais e 15 não verbais), entre 2 e 9 anos, aproximadamente, com diagnóstico médico de TEA, realizado em equipe. Para a análise do desenvolvimento, aplicou-se a escala de desenvolvimento do Perfil Psicoeducacional Revisado (PEP-R), sendo os dados analisados quantitativamente, de forma comparativa e com uso de testes estatísticos, pelo Modelo ANCOVA para análise de covariância, que avaliou a compatibilidade entre os grupos quanto as pontuações obtidas em cada área tendo como covariável a idade cronológica e o Teste t de Student para Amostras Independentes (nível de significância p ≤ 0,001).

**Resultados:**

a pontuação nas diferentes áreas do PEP-R foi superior no grupo verbal, havendo relação, na amostra estudada, entre desenvolvimento da linguagem e de habilidades cognitivas e sócio adaptativas. A comparação entre os grupos indica que o perfil do grupo não verbal se encontra rebaixado, com diferenças estatisticamente significantes nas áreas de imitação, percepção, coordenação motora ampla e fina, integração olho mão, desenvolvimento cognitivo e capacidade cognitiva verbal.

**Conclusão:**

o objetivo de comparar o perfil psicoeducacional de autistas verbais e não verbais foi atingido, apontando diferenças significativas. O perfil dos indivíduos com TEA não verbais analisados na amostra se encontra rebaixado em relação aos verbais. Sugerem-se novos estudos com amostras maiores, faixas etárias delimitadas e com mais testes específicos em cada área do desenvolvimento.

## INTRODUÇÃO

O Transtorno do Espectro Autista (TEA), baseado no Manual Diagnóstico e Estatístico de Transtornos Mentais (DSM-5), é definido como um transtorno do neurodesenvolvimento, caracterizado pelo desenvolvimento atípico e prejudicado com diferentes graus de severidade, resultando no comprometimento das habilidades sociais, habilidades comunicativas verbais e não verbais, além da manifestação de comportamentos e interesses em atividades restritos, com presença de movimentos repetitivos e estereotipados^(1)^. Apresenta origem multifatorial, requerendo então a ação de examinar e compreender todos os aspectos relacionados às questões genéticas familiares, emocionais e sociais das quais o indivíduo faz parte. O delineamento do perfil psicoeducacional da criança com TEA é essencial para o prognóstico, atrelado ao planejamento da intervenção, devendo ser considerados déficits na coordenação motora fina e sensorial, atrasos na linguagem, e deficiência intelectual^(2)^.

As manifestações do TEA iniciam-se muito precocemente e as famílias relatam que grande parte das características são perceptíveis no dia-a-dia da criança e podem se manifestar em maior ou menor grau dependendo da demanda ambiental. O déficit na interação social pode se manifestar mais perceptivelmente quando há aumento do círculo social da criança (como, por exemplo, entrada no ambiente escolar), devido à dificuldade na socialização e o comprometimento do contato com outros interlocutores. Na comunicação, pode ser observado desde atraso do desenvolvimento da fala e da linguagem, até uma fala com restrições de funções comunicativas. Já em relação ao comportamento, pode ser observado o estabelecimento de uma constante rotina, além das estereotipias e comportamentos repetitivos manifestados em graus e formas diferentes individualmente, mas caracterizadas pelas repetições de padrão e aparente ausência de funcionalidade. As consequências do transtorno poderão ser mais leves ou graves, de acordo com o nível de manifestação e funcionamento em que se encontra a pessoa com TEA no momento^(3)^.

A atuação interdisciplinar, desde o diagnóstico até a intervenção, em equipe composta por médico, psicólogo, educadores (pedagogo, psicopedagogo, etc.), terapeuta ocupacional e fonoaudiólogo, se torna indispensável, pois esta ajuda a desenvolver a comunicação verbal e não verbal, socialização, integração sensorial, alfabetização e outras habilidades dependendo do seu grau de comprometimento e da intensidade e adequação do tratamento^(4)^.

No decorrer do desenvolvimento da linguagem em crianças com desenvolvimento típico de linguagem há variações individuais, tanto no processo de aquisição em si quanto na velocidade e qualidade em que a linguagem é adquirida, tratando-se então de um desenvolvimento complexo e dependente de uma gama de fatores, compreendendo desde maturação neuropsicológica, afetividade, desenvolvimento cognitivo, até contextos nos quais a criança está inserida^(5)^.

Além disso, a criança tem todo seu desenvolvimento dependendo não somente de fatores biológicos, mas também de fatores ambientais ou socioambientais (como estimulação, condições socioeconômicas e ambiente familiar). Uma relação quase sempre estabelecida nos estudos da área da linguagem é que o desenvolvimento cognitivo está associado ao desenvolvimento linguístico. Dessa maneira, quanto melhor for o desenvolvimento da linguagem, mais hábeis as crianças serão em comunicar seus pensamentos, sentimentos, ideias, intenções e também compreender os mesmos processos nos outros. De acordo com essa premissa, linguagem e cognição estariam intimamente ligados^(6)^.

Em relação aos TEA, a questão cognitiva não pode ser apenas atrelada com clareza ao quociente intelectual do indivíduo, nem mensurada por meio de testes psicométricos que utilizem a oralidade de forma direta, já que grande parte dos estudos que comparam grupos de pessoas com TEA não verbais e minimamente verbais com grupos verbais não relaciona o quociente intelectual como medida confiável diretamente relacionada ao fato de um indivíduo com TEA ser considerado verbal ou não^(7-11)^.

No entanto, não se pode negar que o desenvolvimento da linguagem e da fala é correlacionado a uma série complexa de habilidades cognitivas, perceptuais e linguísticas cuja gênese está no período pré-verbal. A construção simbólica faz parte das habilidades cognitivas essenciais para a formação do signo linguístico e, consequentemente, da utilização de vocábulos como forma de expressão. Assim, o desenvolvimento do simbolismo está diretamente relacionado ao da linguagem oral^(12)^.

O comprometimento da comunicação nos quadros de TEA afeta tanto as habilidades verbais quanto não verbais, em graus variados, dada a heterogeneidade do quadro. A população com TEA apresenta frequentemente um repertório de elementos verbais (palavras e frases) ausente ou inconsistentemente estabelecido, principalmente em início do desenvolvimento. Algumas crianças não desenvolvem as habilidades de comunicação, com ausência total da fala. Outras apresentam linguagem pouco desenvolvida, podendo ser caracterizada por jargões, ecolalias, inversões pronominais, prosódia anormal e entonação monótona. Aqueles que adquirem habilidades verbais podem demonstrar déficits persistentes em estabelecer conversação, tais como falta de reciprocidade social e de funcionalidade na sua comunicação^(13)^.

Em relação aos estudos da área^(10,11,14-20)^, muitos tendem a adotar definições sobre o que seria considerada uma criança com TEA verbal, minimamente verbal e não verbal^(11)^ e, embora até o momento não exista uma definição compartilhada pela maioria dos autores, dada a heterogeneidade no funcionamento intelectual e nas habilidades linguísticas entre esses indivíduos com TEA, há alguns parâmetros a serem considerados, tanto baseados em observações diretas e aplicação de testes e protocolos de linguagem expressiva e receptiva, quanto em análises de vídeos de situações comunicativas e nos relatos dos pais. Indivíduos com TEA considerados verbais (V) em grande parte das pesquisas são os que produzem frases funcionais variadas em mais de um contexto comunicativo, em seu repertório linguístico de comunicação^(10,14-16)^.

Sobre os indivíduos minimamente verbais (MV), há uma forte discussão na literatura destes se enquadrarem na categoria verbais (V) ou não verbais (NV); muitos autores^(10,14,16,18)^ adotam que indivíduos minimamente verbais são aqueles que utilizam funcionalmente, no mínimo, um pequeno repertório (de 10 a 30) de palavras isoladas em sua comunicação e/ou palavras e frases fixas, em um ou mais contextos comunicativos de interação^(15-17)^. Já indivíduos não-verbais (NV) seriam os que não desenvolveram nenhuma linguagem verbal funcional ou permanecem minimamente verbais, sem expansão de seu repertório comunicativo após 12 anos de idade (início da adolescência), embora uma parte dos autores sugiram até 7 anos de idade^(11,16,18-20)^. Como já citado, não há um consenso entre os pesquisadores da área, o que faz com que - ao se realizarem pesquisas sobre o tema - os autores tenham que se posicionar a respeito de qual definição utilizarão de forma clara, o que nem sempre ocorre, dificultando revisões sistemáticas de literatura com metanálise confiável.

Na perspectiva dos já citados autores^(10,11,18-20)^, a fim de melhor compreender as características clínicas e os correlatos neurobiológicos desse subgrupo (V e MV) de indivíduos com TEA, seria apropriado defini-los com critérios diagnósticos muito rigorosos e que as pesquisas na área especificassem cada vez mais detalhadamente quais os critérios utilizados nas definições de seus grupos experimentais e controle com essas populações.

O Perfil Psicoeducacional Revisado - PEP-R^(21,22)^ foi construído especificamente para avaliar crianças com TEA ou com transtornos correlatos da comunicação. Tal instrumento surgiu em função da necessidade de identificar padrões irregulares de aprendizagem, fornecendo informações valiosas para elaboração de um planejamento psicoeducacional individualizado, segundo os princípios do Modelo TEACCH (Treatment and Education of Autistic and Communication Handicapped Children)^(21)^, sendo que profissionais que trabalham com esse modelo utilizam o PEP-R com frequência em suas avaliações e reavaliações clínicas ou educacionais. No entanto, outras linhas teóricas de base comportamental ou desenvolvimentistas também fazem uso do instrumento. O instrumento já foi traduzido e validado para o Português Brasileiro em vem sendo usado como referência^(22)^ em muitas pesquisas e também clinicamente na área da Saúde e Educação, como uma referência específica de avaliação de desenvolvimento de crianças com TEA^(23-26)^.

A pesquisa brasileira de validação do PEP-R^(22,23)^ apresenta as propriedades psicométricas da versão brasileira do mesmo, que avalia a idade de desenvolvimento em crianças com autismo ou com transtornos correlatos da comunicação. A validade de critério das dimensões da escala de Desenvolvimento e Comportamento foi avaliada administrando-se o PEP-R em 20 crianças com autismo, 20 crianças com Síndrome de Down e 40 crianças com desenvolvimento típico, com idades entre 4 e 9 anos. Foram também administradas as Matrizes Progressivas Coloridas de Raven e a versão brasileira do CBCL (Child Behavior Checklist). As correlações entre o PEP-R e essas medidas foram de 0,54 e 0,39, respectivamente. A fidedignidade entre os avaliadores (W-Kendall) variou entre 0,80 e 0,87 e a consistência interna entre 0,80 e 0,97. A comparação dos escores brutos finais da escala de Comportamento do PEP-R, nos três grupos, confirmou a validade discriminante do instrumento, sendo que o grupo com autismo apresentou maior comprometimento que os demais grupos nas dimensões investigadas.

Em estudo posterior^(25)^ utilizando o PEP-R, verificou-se que em crianças autistas, com idades entre 1 ano e 3 meses e 2 anos e 11 meses, nas áreas de desempenho cognitivo e cognitivo verbal o desempenho das mesmas se encontrou muito rebaixado, provavelmente porque essas áreas englobam habilidades diretamente relacionadas ao repertório de fala.

Os estudos envolvendo o PEP-R^(22-26)^, no geral, apontam os níveis do funcionamento do desenvolvimento e anormalidades no comportamento das crianças, especialmente as com diagnóstico de TEA, permitindo construir planejamentos educacionais que levem em consideração as habilidades adquiridas e as que estão em desenvolvimento. Entender as diferenças e estágio de funcionamento de habilidades relacionadas ao desenvolvimento, em indivíduos verbais e não verbais, permite que profissionais que trabalham com esses indivíduos possam ter uma orientação em suas práticas de intervenção, redirecionando as estratégias e traçando um planejamento diferencial, com prioridades diferentes para cada perfil, com enfoque no aprimoramento de cada habilidade, que consequentemente se torna requisito para o desenvolvimento das demais habilidades^(25)^. Conhecer as limitações de cada perfil, verbal e não verbal, corrobora para implantação de modelos diferenciados de atuação bem como, contribuição para o olhar clínico e considerações em relação ao prognóstico.

Por fim, o objetivo da pesquisa aqui relatada foi comparar o perfil psicoeducacional de crianças com TEA verbais e não verbais. A hipótese do presente era a de que crianças verbais com TEA apresentariam perfil psicoeducacional com desempenho superior em relação aos não verbais, levando em consideração a ordem cronológica de domínio e desenvolvimento de certas habilidades, que ocasionalmente são requisitos para adquirir outras, com enfoque principalmente no que diz respeito a oralidade (relação verbal x não verbal). Os pesquisadores, a todo momento, se preocuparam em especificar quais seriam - na população estudada - as habilidades com desempenho superior de crianças verbais e em quais o desempenho se igualaria ou equipararia aos de crianças não verbais, para melhor compreensão das interpelações entre os vários domínios do desenvolvimento nessa população.

## MÉTODO

O presente estudo foi desenvolvido na Faculdade de Odontologia de Bauru (FOB-USP), da Universidade de São Paulo. Para seu desenvolvimento foram considerados, em todas as suas etapas, os princípios éticos fundamentais que norteiam a pesquisa envolvendo seres humanos, descritos e estabelecidos pela Resolução CNS 466/12 e suas complementares, tendo havido a aprovação do Comitê de Ética em Pesquisa em Seres Humanos da FOB-USP, sob protocolo nº 4.588.003.

Foi realizada a avaliação de 30 indivíduos com diagnóstico clínico médico de TEA, realizado em equipe interdisciplinar, sem quadros associados, respeitando os critérios diagnósticos do Manual Diagnóstico e Estatístico de Transtornos Mentais (DSM-5)^(1)^ e Classificação Estatística Internacional de Doenças e Problemas Relacionados à Saúde (CID-10)^(27)^, vigentes na época da coleta de dados, sendo 15 verbais (Grupo 1) e 15 não verbais (Grupo 2), com intervalo de idade entre 2 anos e 3 meses e 9 anos e 3 meses.

Para definição de quais crianças seriam consideradas verbais e quais seriam consideradas não verbais, embora - como citado na introdução - não haja consenso geral na literatura, na pesquisa aqui apresentada foram considerados os critérios expostos e utilizados por autores relacionados ao referencial teórico utilizado em estudos similares^(10,11,14-20)^ e pela recente revisão de literatura^(11)^ sobre as características clínicas e neurobiológicas de crianças minimamente verbais com TEA. Tal revisão mostra que grande parte dos estudos que comparam grupos de pessoas com TEA não verbais e minimamente verbais com grupos verbais não relaciona o quociente intelectual como medida confiável diretamente relacionada ao fato de um indivíduo com TEA ser considerado verbal ou não^(7-11)^, o que também justifica o motivo desse fator não ter sido considerado na amostra da pesquisa aqui apresentada.

Importante deixar claro que - embora até o momento não exista uma definição compartilhada pela maioria dos autores do que seja considerada uma criança verbal, minimamente verbal ou não verbal na área dos TEA - dada a heterogeneidade no funcionamento intelectual e nas habilidades linguísticas entre esses indivíduos, foram adotadas, por fim, as seguintes definições (baseadas nos relatos dos pais e nos vídeos realizados na coleta de dados) dos grupos de pesquisa:

***Grupo 1*** - nesse grupo, considerado o de “crianças verbais” foram incluídas:

- Criança verbal (V): crianças que produziam frases funcionais variadas em mais de um contexto comunicativo, em seu repertório linguístico de comunicação, no momento da coleta de dados^(10,14-16)^
- Criança minimamente verbal (MV): crianças que usavam funcionalmente, no mínimo, um pequeno repertório (de 10 a 30) de palavras isoladas em sua comunicação e/ou palavras e frases fixas, em um ou mais contextos comunicativos de interação^(10,14-18)^ no momento da coleta de dados.

***Grupo 2*** - nesse grupo, considerado o de “crianças não verbais” foram incluídas:

- Criança não verbal (NV): crianças que não desenvolveram nenhuma linguagem verbal funcional ou permaneceram minimamente verbais, sem expansão de seu repertório comunicativo^(11,16,18-20)^ até o momento da coleta de dados da pesquisa aqui relatada.

Todos os participantes e seus pais e/ou responsáveis preencheram e assinaram o termo de consentimento livre e esclarecido e/ou termo de assentimento livre e esclarecido.

Foi utilizado como instrumento de coleta de dados a Escala de Desenvolvimento do Perfil Psicoeducacional Revisado (PEP- R)^(21),^ versão traduzida e validada para o Português Brasileiro^(22)^ no qual foram analisados os aspectos relacionados ao desenvolvimento e perfil socioeducacional de crianças com TEA, sendo o mesmo aplicado por uma fonoaudióloga habilitada no teste. Todas as aplicações do instrumento foram gravadas.

O PEP-R^(21,22)^ é um instrumento aplicável em crianças de nível pré-escolar, dentro ou abaixo do intervalo de idade entre 2 à 12 anos de idade cronológica, que mensura a idade de desenvolvimento e identifica padrões de aprendizado irregulares e idiossincráticos de crianças com TEA. É um instrumento amplamente utilizado em estudos internacionais e nacionais na área dos TEA, já tendo sido validado para o Português Brasileiro^(22,23)^ e que apresenta propriedades psicométricas que possibilitam a confirmação da validade discriminante do instrumento.

O instrumento PEP-R contempla duas escalas de avaliação, uma relacionada ao Desenvolvimento e outra ao Comportamento. As áreas de desenvolvimento, utilizadas no estudo, são 7, sendo elas: coordenação motora ampla (18 itens), coordenação motora fina (16 itens), coordenação viso motora/integração olho mão (15 itens), percepção (13 itens), imitação (16 itens), desempenho cognitivo geral (26 itens) e cognitivo verbal/linguagem (27 itens), totalizando 131 itens avaliados. As áreas de imitação, linguagem e cognição estão diretamente ligadas ao desenvolvimento global do indivíduo. Para cada área, foi desenvolvida uma escala específica com tarefas a serem realizadas ou comportamentos a serem observados, sendo esse número de itens - como citado - variável por área de desenvolvimento.

O kit para aplicação do PEP-R é constituído por uma série de brinquedos e materiais pedagógicos que foram apresentados para a criança de uma forma lúdica e estruturada como jogo e brincadeira. A aplicação do teste ocorreu na própria instituição em horários pré-selecionados, de acordo com a disponibilidade da família e da criança, individualmente, em uma sala de avaliação disponível no local, ampla, iluminada e silenciosa. A aplicação para cada criança durou uma única sessão, devidamente gravada em vídeo de - no máximo - 90 minutos, com intervalo, caso necessário (tempo mínimo 60 minutos). No decorrer das aplicações estavam presentes a pesquisadora, a fonoaudióloga aplicadora e a criança.

Para melhor aplicação, os itens do teste são dispostos em ordem de acordo com o desenvolvimento, ou seja, das atividades mais simples fáceis para as mais complexas. Para fornecer o comando de execução das atividades, devem ser evitadas instruções verbais padronizadas. No Manual do avaliador PEP- R há algumas técnicas específicas de aplicação, como: predomínio de instruções verbais, com uso de linguagem simplificada de forma a orientar a criança, uso de dicas e alguns gestos para informar aquilo que é pedido a criança, demonstrações do modo como a atividade deve ser realizada e direcionamento físico durante as tarefas, fornecendo ajuda com os materiais, movimentando as mãos da criança para os mesmos. Ao final da sessão, a pontuação/escores obtidos são distribuídos entre as áreas de desenvolvimento e comportamento (no caso da presente pesquisa, foi utilizada somente a área de desenvolvimento).

Em relação a pontuação, na escala de desenvolvimento a mesma é classificada de 3 modos diferentes, sendo eles: Passou (P), quando a criança executar a tarefa de forma correta (a qual foi atribuído 1 ponto na pesquisa aqui relatada), ou seja, com sucesso, Emergente (E), quando a criança demonstra algum tipo de conhecimento para executar a tarefa, porém não consegue realizá-la totalmente ou completá-la (a qual é atribuído 0,5 ponto) e Reprovado (R), quando a criança realmente não demonstra conhecimento para executar a atividade mesmo após inúmeras demonstrações e consequentemente não realiza a tarefa (não há pontuação).

Os dados foram computados e analisados de forma quantitativa pelo modelo ANCOVA, que avaliou a compatibilidade entre os grupos quanto as pontuações obtidas em cada área tendo como covariável a idade cronológica. Essa análise permite que seja feito um “ajuste” do efeito de uma variável de resposta que sofreu influência de uma variável ou uma causa de variação não controlada (como, por exemplo, a ampla faixa etária). Além disso, também foi utilizado o Teste t de Student para Amostras Independentes. Como nível/parâmetro de significância considerou-se p ≤0,001.

A análise de covariância realizada pelo modelo ANCOVA é utilizada justamente quando em um experimento se encontra a dificuldade de obter unidades experimentais semelhantes, como no caso da presente pesquisa. A ANCOVA pode incluir uma ou mais variáveis quantitativas que estão relacionadas ao desfecho de interesse e essas variáveis são incluídas na análise por conta da influência que elas possuem sobre o desfecho sendo conhecidas como covariáveis. Através do uso de covariáveis no modelo de ANCOVA, pode-se: eliminar o erro sistemático que ocorre fora do controle do pesquisador e que corriqueiramente pode enviesar os resultados e explicar as diferenças nas respostas devido a características dos respondentes.

## RESULTADOS

O PEP-R possui parâmetros psicométricos para traçar a idade de desenvolvimento das crianças, conforme a pontuação obtida em cada área da escala de desenvolvimento. É importante ressaltar que, ao analisar o público total da amostra, foi evidenciada a diferença da idade cronológica entre o grupo verbal e não verbal, com impossibilidade de pareamento. Foi utilizado o Teste t de Student para Amostras Independentes, para comparar os dois grupos quanto à idade cronológica visando confirmar a diferença significativa entre as idades dos grupos que confirmou a hipótese de que estatisticamente há diferença significativa entre as idades (t=-2,503 e p=0,018). Com isso, foi evidenciada uma limitação na análise dos resultados do estudo e, para que houvesse uma correção deste evento, para comparação entre os grupos, foi utilizada a Análise de Covariância tendo como covariável a idade cronológica, como explicado no item Método da pesquisa.

Na Tabela 1 estão expressas as idades cronológicas e idades de desenvolvimento de cada participante. Vale ressaltar que a idade cronológica e a idade de desenvolvimento atribuída pelo PEP-R estão representadas numericamente por meses. Assim é possível observar que todas as crianças avaliadas, tanto verbais quanto não verbais, possuem idade de desenvolvimento inferior à idade cronológica, sendo que a diferença de idade cronológica e idade de desenvolvimento no grupo verbal variou de 19 a 108 meses (aproximadamente 1 ano e 6 meses a 9 anos), enquanto que no grupo não verbal variou de 18 a 92 meses (aproximadamente 1 ano e 5 meses a 7 anos e 6 meses).

**Tabela 1:** Diferença entre Idade cronológica e Idade de desenvolvimento traçada pelo PEP-R no Grupo 1 e Grupo 2

Grupo Verbal (G1)				Grupo Não Verbal (G2)			
Participante	Idcrono	Idpep	Diferença	Participante	Idcrono	Idpep	Diferença
1	66	42	24	1	41	9	32
2	81	45	36	2	27	7	20
3	85	60	25	3	54	22	32
4	57	20	37	4	46	12	34
5	41	22	19	5	37	12	25
6	66	22	44	6	29	9	20
7	132	24	108	7	109	17	92
8	64	41	23	8	60	18	42
9	48	20	28	9	49	16	33
10	50	22	28	10	45	22	23
11	46	22	24	11	33	4	29
12	53	32	21	12	68	16	52
13	65	28	37	13	69	18	51
14	83	55	28	14	24	6	18
15	111	36	75	15	33	14	19
Média	69,87	25,20	44,13	Média	48,27	21,27	27,00
Desvio Padrão	25,73	8,86	26,24	Desvio Padrão	21,96	8,48	23,66

**Teste estatístico adotado**: Modelo ANCOVA (análise de covariância) e Teste t de Student para Amostras independentes, com nível de significância p ≤ 0,001.

**Legenda:** Idcrono = Idade cronológica em meses; Idpep = Idade de desenvolvimento traçada pelo protocolo PEP-R em meses; Diferença = diferença entre idade cronológica e idade traçada pelo PEP-R em meses.

Na Tabela 2 foi observado que os valores de *p da análise de covariância realizada foram significativos em todas as áreas do protocolo PEP-R, confirmando a hipótese de que o desempenho de indivíduos com TEA verbais foi superior ao dos não verbais, em todas essas áreas. Além disso, em uma observação geral, fica evidente como as médias gerais em todas as áreas de desenvolvimento são maiores no grupo verbal em relação ao grupo não verbal, com valor de *p significativo na análise de covariância (Tabelas 2 a 5).

**Tabela 2:** Médias e análise de covariância expressa por p valor com base na hipótese alternativa (H) de que o desempenho do Grupo Verbal (G1) é superior ao do Grupo Não Verbal (G2)

Item avaliado	Média				P valor
	**Verbal (G1)**		**Não Verbal (G2)**		H=G1>G2
	**Vobt**	**Vmáx**	**Vobt**	**Vmáx**	
Imitação	10,27	16	1,67	16	**0,000011**
Percepção	10,73	13	6,07	13	**0,000088**
Mfino	9,13	16	4,20	16	**0,009853**
Mgrosso	13,40	18	6,60	18	**0,000083**
Olho mão	8,60	15	3,73	15	**0,008168**
Cogeral	12,67	26	2,33	26	**0,000403**
Coverbal	9,87	27	1,07	27	**0,000408**
Ptotal	74,67	131	25,67	131	**0,000049**

**Teste estatístico adotado** = Modelo ANCOVA (análise de covariância), com nível de significância p ≤ 0,001.

**Legenda:** Vobt = Valor obtido no item pela criança no momento da coleta de dad**os; Vmáx** = Valor máximo possível no item avaliado, segundo o protocolo; Mfino = Motor Fino**; Mgrosso** = motor grosso; Cogeral = cognitivo geral; Coverbal = cognitivo verbal/linguagem; Ptotal = pontuação total; H=G1>G2 = hipótese alternativa (desempenho do grupo verbal superior ao não verbal).

**Tabela 5:** Comparação do desempenho do Grupo Verbal (G1) X Grupo Não Verbal (G2) nas áreas Desempenho Cognitivo Geral (C. Geral) e Desempenho Cognitivo Verbal (C. Verbal) do protocolo PEP-R

Participante	Grupo Verbal		Grupo Não Verbal	
	Cognitivo Geral	Cognitivo Verbal	Cognitivo Geral	Cognitivo Verbal
1	17	19	2	1
2	17	13	0	1
3	26	23	7	2
4	4	5	1	1
5	4	4	1	0
6	6	3	0	0
7	12	3	4	1
8	18	10	2	1
9	4	5	2	4
10	12	5	7	2
11	4	2	0	0
12	14	10	2	1
13	10	13	6	1
14	23	18	1	0
15	19	15	0	1
Média	12,67	9,87	2,33	1,07
Desvio Padrão	7,29	6,7	2,49	1,03
Intervalo (vmín - vmáx)	4-26	2-23	0-7	0-4
			(p) C. Geral	**0,000403**
			(p) C. Verbal	**0,000408**

**Teste estatístico adotado**: Modelo ANCOVA (análise de covariância) e Teste t de Student para Amostras independentes, com nível de significância p ≤ 0,001.

**Legenda:** Intervalo (vmín - vmáx) **=** valores mínimos e máximos de pontuação obtido em cada área avaliada.

Analisando isoladamente a variável Imitação (Tabela 3), ressalta-se que essa área contém atividades que avaliam a habilidade da criança de imitar de forma verbal ou motora; no geral, a pontuação na imitação de indivíduos verbais variou de 2 a 16 pontos, enquanto que a dos não verbais variou de 0 a 8 pontos. Já a Percepção, também demonstrada na Tabela 3, diz respeito a área que realiza a checagem do funcionamento das modalidades sensoriais, visual e auditiva. Os resultados mostram que, no geral, a pontuação do grupo verbal foi superior (variando de 9 a 13 pontos) à do grupo não verbal (variação de 2 a 11 pontos).

**Tabela 3:** Comparação do desempenho do Grupo Verbal X Grupo Não Verbal nas áreas imitação e percepção do protocolo PEP-R

Participante	Grupo Verbal		Grupo Não Verbal	
	Imitação	Percepção	Imitação	Percepção
1	15	12	1	3
2	15	10	0	3
3	16	13	8	10
4	6	9	2	5
5	7	9	2	8
6	9	9	1	3
7	13	10	1	8
8	13	11	1	9
9	2	10	1	6
10	5	11	7	7
11	5	10	0	2
12	11	12	0	7
13	9	11	1	11
14	15	13	0	3
15	13	11	0	6
Média	10,27	10,73	1,67	6,07
Desvio Padrão	4,46	1,33	2,47	2,84
Intervalo (vmín - vmáx)	2-16	9-13	0-8	2-11
			(p) imitação	**0,000011**
			(p) percepção	**0,000088**

Teste estatístico adotado - Modelo ANCOVA (análise de covariância) e Teste t de Student para Amostras independentes, com nível de significância p ≤ 0,001.

**Legenda:** Intervalo (vmín - vmáx) **=** valores mínimos e máximos de pontuação obtido em cada área avaliada.

Nas áreas Coordenação Motora Ampla e Coordenação Motora Fina do protocolo PEP-R há atividades como andar sozinho, subir escadas e se equilibrar sobre um dos pés (no motor amplo) e cortar usando tesoura e desrosquear uma tampa (no motor fino). A Tabela 4 apresenta os resultados obtidos nas áreas motoras, que revelam ser as pontuações do grupo verbal maiores que as do não verbal, sendo que - na coordenação motora ampla -os resultados dos indivíduos com TEA verbais variaram de 9 a 18 pontos e o dos não verbais de 2 a 12 pontos e - na coordenação fina - os resultados dos verbais variaram de 5 a 16 pontos e dos não verbais de 0 a 10 pontos.

**Tabela 4:** Comparação do desempenho do Grupo Verbal X Grupo Não Verbal nas áreas Coordenação Motora Fina, Coordenação Motora Ampla e Integração Olho-Mão do protocolo PEP-R

Participante	Grupo Verbal			Grupo Não Verbal		
	Motor Fino	Motor Amplo	Olho-Mão	Motor Fino	Motor Amplo	Olho-Mão
1	9	14	10	4	3	1
2	13	17	13	1	2	1
3	16	18	15	10	11	8
4	6	12	6	7	9	3
5	5	12	5	1	4	2
6	11	9	4	1	6	0
7	6	13	4	2	10	4
8	14	17	10	10	7	6
9	8	10	6	3	7	5
10	6	10	5	10	12	8
11	5	13	6	0	2	0
12	10	15	8	4	7	6
13	6	13	10	7	10	7
14	14	18	15	0	2	1
15	8	10	12	3	7	4
Média	9,13	13,4	8,6	4,2	6,6	3,73
Desvio Padrão	3,68	3,04	3,85	3,69	3,4	2,84
Intervalo (vmín-vmáx)	5-16	9-18	4-15	0-10	2-12	0-8
					(p) Motor Fino	**0,009853**
					(p) Motor amplo	**0,000083**
					(p) Olho-Mão	**0,008168**

**Teste estatístico adotado**: Modelo ANCOVA (análise de covariância) e Teste t de Student para Amostras independentes, com nível de significância p ≤ 0,001.

**Legenda:** Intervalo (vmín - vmáx) = valores mínimos e máximos de pontuação obtido em cada área avaliada.

A Tabela 4 também aponta as pontuações referentes à área Integração Olho Mão, sendo esta composta por atividades como escrever em papel, colorir dentro de áreas limitadas, desenhar e copiar figuras e empilhar vários blocos. A pontuação obtida no grupo de crianças verbais (variando de 4 a 15 pontos) foi superior à obtida pelos não verbais (variando de 0 a 8 pontos).

As áreas Desempenho Cognitivo Geral e Desempenho Cognitivo Verbal/Linguagem no instrumento PEP-R possuem respectivamente pontuação máxima de 26 e 27 pontos. Essas áreas são centradas na cognição geral e verbal/linguagem. As atividades de desempenho cognitivo geral requerem alguma compreensão receptiva da linguagem para execução bem-sucedida. A diferença principal entre as duas áreas é que os itens de desempenho cognitivo geral são atividades de execução que não requerem linguagem expressiva, enquanto os itens cognitivos verbais solicitam uma resposta verbal. Na Tabela 5 é possível observar a diferença significativa no desempenho de indivíduos do grupo verbal e não verbal, em ambas as áreas. No desempenho cognitivo geral, os resultados dos indivíduos verbais variaram de 4 a 26 pontos e os dos indivíduos não verbais de 0 a 7 pontos. Já no desenvolvimento cognitivo verbal, os resultados variaram de 2 a 19 pontos para o grupo verbal e de 0 a 4 pontos para o não verbal.

## DISCUSSÃO

De forma geral, comparou-se o perfil psicoeducacional de crianças com TEA verbais e não verbais, confirmando a hipótese principal da pesquisa de que crianças verbais com TEA apresentaram - na amostra analisada - desempenho superior em relação aos não verbais.

A visão desenvolvimentista em relação às áreas de domínio avaliadas por itens vinculados a comportamentos específicos, considera a ordem cronológica de domínio e desenvolvimento de certas habilidades que ocasionalmente são requisitos para adquirir outras, com enfoque principalmente no que diz respeito à oralidade (relação verbal x não verbal).

A principal contribuição que os resultados da pesquisa aqui apresentada foi analisar e especificar quais seriam - na população estudada - as habilidades com desempenho superior de crianças verbais e em quais o desempenho se igualaria ou equipararia aos de crianças não verbais, para melhor compreensão das interpelações entre os vários domínios do desenvolvimento nessa população, com impactos futuros no delineamento de intervenções clínicas e escolares.

No entanto, há importantes limitações a serem discutidas. Num primeiro momento, é necessário considerar que alguns dos indivíduos do grupo de crianças verbais apresentavam idade cronológica superior aos integrantes do grupo não verbal, o que pode colaborar para a referida diferença de valores de pontuação em muitos itens avaliados, embora para isso tenham sido aplicados testes estatísticos que amenizassem essa diferença na análise comparativa.

Como o intervalo de idade da presente pesquisa é de 2 anos e 3 meses a 9 anos e 3 meses (isto é, entre 27 e 111 meses) tem-se que, quanto maior a idade da criança, melhor desempenho ela tende a ter referente a um maior e mais efetivo uso de comportamentos comunicativos, como o encontrado em estudos da área da linguagem^(6-8)^. O fato de que, nos resultados da pesquisa aqui apresentada, a idade das crianças do grupo verbal variar de 3 anos e 5 meses a 9 anos e 3 meses (41 a 101 meses) e do grupo não verbal variar de 2 anos e 3 meses a 9 anos e 1 mês (27 a 109 meses), pode ter sido um fator de influência nos resultados, mesmo havendo sido realizada a análise de covariância. Esta é uma importante limitação do estudo, pois a abrangência e agrupamento das idades dificultou que os resultados fossem pareados igualmente baseado no pareamento por idades um a um e não grupo a grupo.

As definições de quais seriam consideradas crianças verbais, minimamente verbais e não verbais são fatores relevantes a serem considerados na análise e discussão dos resultados, pois há uma limitação no uso dessas definições, até mesmo por não ser um critério objetivo, embora na pesquisa aqui apresentada tenham sido pautadas em vários renomados autores da área, que inclusive discutem o tema^(10-20)^. Na perspectiva dos já citados autores, a fim de melhor compreender as características clínicas e os correlatos neurobiológicos do subgrupo verbal (V e MV) de indivíduos com TEA, seria apropriado defini-los com critérios diagnósticos muito rigorosos e que as pesquisas na área especificassem cada vez mais detalhadamente quais os critérios utilizados nas definições de seus grupos experimentais e controle com essas populações. Por isso, apresentamos claramente a definição de cada um dos grupos de pesquisa, de forma a que a análise fosse feita à luz das mesmas.

Optou-se pela descrição e discussão de cada área de domínio avaliado, dada à escassez de estudos descritivos nesse formato e para que as pessoas que trabalham na área dos TEA possam ter dados para realizar - ao optar por direcionar sua atuação em abordagens similares à aqui apresentada - uma análise por área com as possíveis relações entre os domínios que queira enfocar em futuras avaliações e intervenções com a população-alvo com a qual atua. Exemplificando, uma fonoaudióloga pode estar mais atenta em alguns de seus pacientes nas relações entre a imitação e o cognitivo-verbal (principalmente em crianças em idade pré-escolar ou em nível pré-verbal) e, em outros momentos ou para outros pacientes (em época pré-alfabetização, por exemplo), seu foco pode ser na percepção, coordenação motora fina e integração olho mão. Sendo assim, a contribuição dos achados aqui expostos é permitir um olhar diferenciado - com o uso do mesmo instrumento - área a área, em duas ou mais áreas ou no perfil psicoeducacional geral proporcionado para os dois grupos avaliados (verbal e não verbal).

A área de imitação do PEP-R, que é responsável por avaliar a capacidade da criança de imitar algo de forma verbal ou motora, é uma área de suma importância para o desenvolvimento de indivíduos com TEA, por causa da relação fundamental entre imitação e linguagem e aprendizagem, visto que para poder aprender palavras, a criança deve ter as habilidades básicas necessárias para imitar, como atenção conjunta e compartilhada, entre outras^(2,5)^. A imitação também tem um papel fundamental na socialização, pois através dela as crianças aprendem estratégias de comportamento, cooperação, coordenação e resposta frente a interações com outras pessoas.

Os itens da área Imitação do PEP-R incluem a imitação de movimentos amplos do corpo, imitação do uso de certos materiais e imitação de sons e palavras, todos demonstrados pelo avaliador com posterior solicitação direta de imitação. Indivíduos com TEA não verbais, por terem dificuldade em demonstrar ou compreender comportamentos verbais (limitação de linguagem receptiva / compreensiva), podem consequentemente apresentar prejuízos quanto à capacidade de imitar principalmente sons e palavras. Tal fato pode ter impactado o desempenho do grupo não verbal nessa área. Do mesmo modo, crianças do grupo verbal que fazem uso mais efetivo das competências comunicativas receptivas e expressivas acabam por manifestar maior facilidade em desempenhar a função de imitação, o que justificaria a pontuação superior do grupo verbal em relação ao grupo não verbal na pesquisa descrita.

Um estudo^(15)^ visando analisar habilidades comunicativas de autistas não-verbais também apontou déficits de desempenho, como diminuição na intensidade e frequência de alguns comportamentos adaptativos, diferença do vocabulário receptivo oral, pragmática com alterações significantes no total de atos comunicativos e atos comunicativos por minuto, redução dos atos comunicativos na imitação, e meio comunicativo com predomínio do meio gestual, sendo os dados sobre perfil pragmático corroborados por outras pesquisas^(6,8,14,16)^. 

Já em relação à Percepção, área que examina a capacidade de selecionar e organizar um estímulo recebido, ressalta-se que crianças com TEA com frequência apresentam dificuldade em armazenar e integrar as informações sensoriais, por isto é importante se atentar às limitações de desempenho nessa área. No PEP-R^(21,22)^, por conta destas dificuldades perceptuais encontradas em indivíduos com TEA, a maioria dos itens de percepção foi formulado para crianças mais novas, sendo que as atividades incluem acompanhar visualmente bolinhas de sabão, observar figuras de um livro, e se orientar de acordo com sons. Considerando os resultados obtidos na área de percepção na pesquisa aqui apresentada, visualiza-se que - mesmo assim - a pontuação geral do grupo verbal comparada a do grupo não verbal foi superior, tendo o grupo de crianças não verbais apresentado maiores dificuldades na realização dos itens nessa área.

Quanto às áreas motoras, sabe-se que a capacidade motora ampla ou global está relacionada ao controle global do corpo, sendo dela a responsabilidade da manutenção do equilíbrio estático e dinâmico e postura corporal. Na infância, essa é a primeira motricidade a ser aperfeiçoada, visto que ela permite as maiores movimentações do corpo, e esses movimentos requerem a participação de muitos músculos do corpo para serem realizados. Além disso, há uma tendência de uma diminuição nas habilidades sociais nos indivíduos que apresentam prejuízo motor amplo grave^(16,28,29)^.

Partindo do pressuposto de que o prejuízo na habilidade motora ampla gera a diminuição de habilidades sociais, isso consequentemente poderá interferir na comunicação oral visto que as habilidades sociais incluem a comunicação. Este fato poderia justificar a pontuação superior na área motora ampla de autistas verbais em relação a autistas não verbais nos resultados dessa pesquisa. Ao avaliar quantitativamente a comparação entre o desempenho motor amplo dos grupos verbal e não verbal, foi observada diferença estatisticamente significativa entre os grupos. Dessa forma, tem-se que o desempenho na área motora ampla do grupo verbal foi superior ao do grupo não verbal.

Em um determinado estudo^(30)^, autores afirmaram que as dificuldades motoras podem interferir em habilidades subsequentes de linguagem expressiva. Esse poderia ser uma das justificativas para a pontuação inferior do grupo não verbal nesta área, ou seja, poderia indicar inter-relação entre: habilidades motoras menos desenvolvidas, maior comprometimento na aquisição de linguagem e maiores dificuldades no desenvolvimento da linguagem oral. Poucos estudos relacionam o desenvolvimento das habilidades motoras amplas ao desenvolvimento exclusivo de linguagem expressiva oral. Desta forma, sugerem-se novos estudos para aprofundamento da questão.

Por outro lado, a capacidade motora fina^(28-30)^ se relaciona pontualmente à execução de movimentos de maior precisão, composta por movimentos mais refinados, requerendo e resultando de um maior controle de músculos específicos. É importante observar que, quanto maior o grau de complexidade a execução de uma atividade exigir, maior sobrecarga de trabalho o sistema cognitivo terá e mais a atuação precisa do sistema nervoso central será exigida.

As atividades da área motora fina que constam no PEP-R^(21,22)^ incluem desatarraxar uma tampa, cortar usando tesoura, e enfiar contas em um barbante. Estas atividades são normalmente dominadas nos primeiros 3-4 anos de vida. Logo, crianças com esse intervalo de idade ou inferior ao mesmo, ainda estão em fase de aquisição dessas habilidades. Nos resultados da pesquisa aqui apresentada, é possível visualizar que, no grupo não verbal, parte dos indivíduos possui idade até 4 anos e isso pode ter contribuído para que a pontuação neste item não tenha sido tão alta quanto a dos indivíduos do grupo verbal (que em sua grande parte possuem idade cronológica maior).

Um estudo realizado em crianças de 14 e 15 meses^(28)^ apontou que a coordenação motora fina pode estar diretamente relacionada ao desenvolvimento da linguagem, pelo fato de as habilidades motoras finas facilitarem a interação entre físico e social, possibilitando então o desenvolvimento expressivo da linguagem. Este fato contribuiria para explicação do desempenho superior de autistas verbais (que possuem linguagem expressiva oral) em relação aos não verbais na vertente motora fina. No entanto, o fato de alguns indivíduos da amostra do grupo não verbal ainda estarem dentro do período de domínio das habilidades motoras finas, cria um impasse na conclusão do desempenho superior do grupo verbal em comparação ao não verbal. Desta forma, pela pesquisa aqui apresentada, apesar do valor de *p ter sido significativo, pelas limitações do estudo relacionadas à idade cronológica divergente entre os grupos, pelo protocolo PEP-R^(21,22)^ e suas provas de avaliação para a vertente motora fina, não se pode afirmar que ser ou não verbal influencia na capacidade motora fina na amostra analisada.

Quanto à Integração Olho Mão, que diz respeito as habilidades viso motoras, estudos^(29)^ revelam que crianças com TEA apresentam características motoras desviantes quanto aos padrões típicos de desenvolvimento desde o nascimento. Déficits motores começam a se manifestar precocemente na vida da criança, antes dos 3 anos. Por ter a mesma dependência e inter-relação que as áreas motoras fina e ampla, as justificativas para que o desempenho de autistas verbais tenha sido superior ao de autistas não verbais na vertente integração olho mão se pautam nas mesmas relatadas nas áreas motoras fina e ampla^(28-30)^. Desta forma, nos resultados da pesquisa aqui apresentada, evidencia-se o desempenho superior de indivíduos com TEA verbais em relação aos não verbais nesse item.

Já em referência às áreas de Desenvolvimento Cognitivo e Cognitivo Verbal, tanto na área de desenvolvimento cognitivo do PEP-R^(21,22)^, que não requer linguagem expressiva oral, quanto na área cognitiva verbal, que solicita uma resposta verbal, a pontuação obtida pelo grupo verbal foi superior à obtida pelo grupo não verbal. Estudos^(6,8,14,16)^ envolvendo o uso interativo da comunicação em crianças autistas verbais e não verbais revelaram que, quanto à utilização dos meios comunicativos pelas crianças autistas, observa-se que tanto as crianças autistas não verbais como as verbais fazem grande uso do meio gestual para se comunicarem. No grupo das crianças autistas verbais, em comparação às não verbais, foi observada maior utilização do meio verbal e menor utilização do meio vocal, como observado em crianças com desenvolvimento típico, o que corrobora os achados da pesquisa citada.

A área de desenvolvimento cognitivo do PEP-R^(21,22)^ possui atividades que requerem principalmente a compreensão receptiva da linguagem para execução bem-sucedida das mesmas. Estudos na área da linguagem relacionam o desenvolvimento da linguagem e o desenvolvimento de outras habilidades cognitivas e sócio adaptativas. Os resultados aqui apresentados evidenciam que a análise por covariância confirmou a hipótese de desempenho superior do grupo verbal em relação ao grupo não verbal. Sabe-se que a cognição de indivíduos com TEA apresenta grande variabilidade^(6,7)^. Como as provas de desenvolvimento cognitivo do PEP-R^(21,22)^ focam principalmente em habilidades compreensivas receptivas, os resultados alcançados pela pesquisa aqui apresentada indicam que, no grupo de indivíduos com TEA não verbal, há um maior comprometimento das capacidades de compreensão e recepção e não somente da expressão (verbal). Mesmo assim, são necessários estudos que visem investigar essas informações com maior detalhamento e aprofundamento.

Em relação a área de Cognição Verbal, numa análise visual inicial dos resultados referentes a essa área, já é possível observar a grande diferença das pontuações do grupo verbal para o grupo não verbal. Este fato já era esperado e pode ser explicado pelo fato de que a maioria das provas de cognição verbal do PEP-R^(21,22)^ necessita de uma resposta verbal. Como os autistas considerados verbais possuem maior domínio da linguagem oral^(16)^, justifica-se o fato de a pontuação deste grupo ter sido significativamente superior à do grupo não verbal, justamente por conta de indivíduos não verbais que compuseram a amostra apresentarem dificuldades em relação a aquisição da linguagem oral até o momento da aplicação da coleta de dados da pesquisa.

## CONCLUSÃO

O PEP-R^(21,22)^ aponta as principais áreas consideradas primordiais ao desenvolvimento de crianças com TEA em seu perfil psicoeducacional, como habilidades de Imitação, Percepção, Motora Fina, Motora Ampla, Coordenação Olho Mão, Desempenho Cognitivo e Cognitivo Verbal. Os achados deste estudo permitem concluir que o perfil socioeducacional dos indivíduos com TEA não verbais analisados na amostra se encontra rebaixado em relação ao perfil de indivíduos com TEA verbais, em todas as áreas do desenvolvimento avaliadas pelo instrumento.

Dessa forma, o objetivo do estudo de comparar o perfil socioeducacional de autistas verbais e não verbais foi atingido e apontou diferenças importantes nas áreas de imitação, percepção, coordenação motora ampla e fina, integração olho mão, desempenho cognitivo global e verbal entre crianças verbais e não verbais com diagnóstico de TEA. Ressalta-se, no entanto, que o estudo possui uma importante limitação em relação ao não pareamento por idades, que pode ter interferido diretamente nos resultados. Sendo assim, sugere-se a elaboração de novos estudos que avaliem de forma mais aprofundada essas áreas em busca de afunilar e detalhar a influência dessas capacidades em indivíduos com TEA verbais e não verbais, com definições claras dos critérios de diferenciação dos grupos, para que a relação comparativa de desempenho entre verbal x não verbal se paute em resultados comprovados cientificamente e não apenas no uso de senso comum em relação à superioridade de um grupo em relação ao outro vinculado à oralidade.

Embora os achados sejam relevantes, já que há poucos estudos que avaliam a comparação do desempenho e das diferenças cognitivas e intelectuais de indivíduos com TEA verbais e não verbais, faz-se necessário a continuidade do estudo entre esses grupos, com aplicação de outros testes e maior número amostral buscando investigar outros aspectos do desenvolvimento e comportamentais destes indivíduos. Reforça-se a importância do pareamento por idades em pesquisas envolvendo grupos verbais e não verbais.
